# Novel piston technique versus Ilizarov technique for the repair of bone defect after lower limb infection

**DOI:** 10.1186/s13018-021-02844-1

**Published:** 2021-12-04

**Authors:** Jiafei Du, Zifei Yin, Pengfei Cheng, Pei Han, Hao Shen

**Affiliations:** 1 Orthopaedic Department, Shanghai Jiao Tong University Affiliated Sixth People’s Hospital, Shanghai, China; 2 Joint Department, Kunshan Hospital of Traditional Chinese Medicine affiliated to Nanjing University of Traditional Chinese Medicine, Jiangsu, China; 3 Orthopaedic Department, Jinjiang Municipal Hospital, Fujian, China

**Keywords:** Ilizarov technique, Piston technique, Masquelet, Induced membrane, Bone defect, Lower limb infection

## Abstract

**Background:**

We aimed to compare the effectiveness and complications of a novel piston technique versus the Ilizarov technique for the repair of bone defects after lower limb infection.

**Patients and methods:**

We retrospectively reviewed 41 patients who had been treated at our department for lower extremity bone defects following osteomyelitis. There were 38 men and three women with a mean age of 43.41 (range, 12–69 years). The infected bone defects involved 36 tibias and five femurs. The piston technique (PT, group A) was used in 12 patients and the Ilizarov technique (IT, group B) in 29 patients. The mean follow-up period was 28.50 months (PT) and 29.90 months (IT). The modified Application of Methods of Illizarov (ASAMI) criteria was used to evaluate bone healing and functional recovery.

**Results:**

Complete eradication of the infection and union of docking sites were accomplished in both groups. The mean external fixator index (EFI) was 42.32 days/cm in group A versus 58.85 days/cm in group B (*p* < 0.001). The bone outcomes were similar between groups A and B (*p* = 0.558) (excellent [9 vs. 19], good [3 vs.10]); group A showed better functional outcomes than group B (*p* < 0.05) (excellent [7 vs. 6], good [4 vs. 12], fair [0 vs. 10] and poor [1 vs. 1]). Pain was the most common complaint during follow-up, and group A had fewer cases of pin tract infection (1 vs. 6), adjacent joint stiffness (3 vs. 8), and delayed healing of the joint (0 vs. 3).

**Conclusions:**

Satisfactory bone healing can be achieved by using both PT and IT, although PT demonstrated better functional results, lower EFI, and allowed early removal of the external fixation. We found that this novel piston technique can improve the comfort of patients, reduce the incidence of complications, and provide rapid and convenient rehabilitation.

**Supplementary Information:**

The online version contains supplementary material available at 10.1186/s13018-021-02844-1.

## Introduction

Infectious bone defects of the lower extremity pose a huge challenge for doctors in clinical practice due to their complicated treatment and unfavorable prognosis. Common causes include acute bone loss, surgical removal of dead or sclerotic bone after infection and ischemic atrophy of nonunion sites [1, 2]. There are numerous options for reconstruction, including Ilizarov distraction osteogenesis [3–6], Masquelet technique [7], allogeneic bone grafts [8] and vascularized autogenous bone grafts (such as ribs, ilium, and fibula) [9]. New tissue engineering techniques [10] are also emerging in the field of bone defect treatment.

The Ilizarov bone transport technique is widely regarded as the standard surgical method for the treatment of bone defects [3] and is used to treat nonunion, osteomyelitis, deformity, traumatic bone defects, and unequal length of the lower extremities. Although the Ilizarov technique can achieve satisfactory results in most cases, one of the most common problems associated with this method is that the external fixator needs to be fixed for a long time until the new bone is completely ossified, which involves many complications, including pin trajectory infection, loosening of the retractor, persistent pain, joint stiffness, angled tilt, refracture and delayed union [11, 12]. In recent years, a high value has been placed on the Masquelet technique [13, 14], and it is widely used. It is, a two-stage surgical technique, which involves debridement and filling of the bone defect with cement spacer in the first stage to control postoperative infection, followed by bone remodeling by filling with cancellous bone after removing the cement inside the so-called induced membrane in the second stage. Due to the risk of residual infection after the first-stage operation for infectious bone defects, a mixture of bone cement and antibiotics has been widely used [15–17]. There are several clinical series reports [3–7, 13, 18] on the reconstruction of bone defects using the Masquelet technique. However, in some cases, several bone grafts are needed, and donor site injury can also occur. To overcome these technical limitations, our department used the Masquelet technique combined with the Ilizarov bone transport technique, which is called the piston technique, for the first time to treat bone defects after lower extremity infection. The piston technique involves a similar procedure in the first stage as the Masquelet technique, and we removed the bone cement without disturbing the induced membrane. External fixation was then used to perform distraction osteogenesis in the second stage. This is expected to reduce the time of external fixation and avoid bone grafting.

To the best of our knowledge, there are relatively few reports describing the outcomes of the piston technique for the treatment of infected bone defects. Therefore, here we have described this piston technique, and compared it with the Ilizarov bone transport technique for the treatment of bone defects after lower extremity infection. We aimed to determine whether this technique could reduce the fixator duration time in patients and the risk of complications.

### Patients and methods

This retrospective study was reviewed and approved by the institutional review board. All procedures were performed in compliance with the Declaration of Helsinki. Informed consent for participation was obtained from all participants.

We retrospectively analyzed the outcomes of the piston technique (PT) or Ilizarov technique (IT) for the treatment of bone defects after lower limb infection between 2013 and 2018. The inclusion criteria for this study were: (1) osteomyelitis diagnosed by bacteriology or histology following intraoperative biopsy based on clinical manifestations and imaging; (2) patients treated with the PT or IT; (3) bone defect > 2.5 cm after debridement; and (4) age < 70 years. Exclusion criteria were as follows: (1) patients with bone tumors, rheumatic diseases and metabolic bone diseases; (2) patients who withdrew from treatment early or did not complete the treatment; or (3) patients who could not be followed up.

Finally, 29 and 12 patients in the IT and PT groups, respectively, were enrolled in the study. There were 38 men and three women and the bone defects included 36 tibias and five femurs. The basic information of the patients is shown in Additional file 1: Tables S1 and S2. The variables included sex, age, site, organism, type of external fixator, previous operations, comorbidities, soft tissue defects and bone defects.

### Surgical techniques

**Fig. 1.**
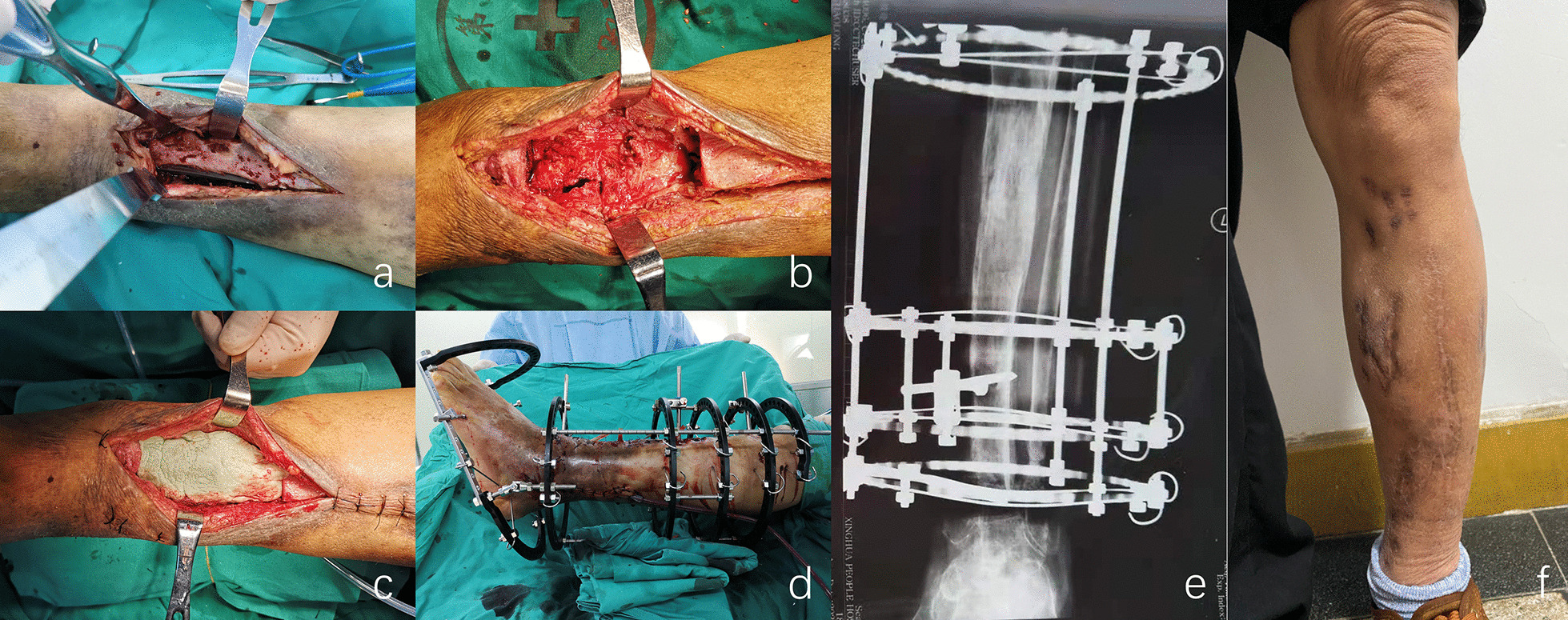
54-year-old man with left tibia osteomyelitis, having a bone defect length of 9.8 cm, was performed with Piston technique. **a**, **b** During debridement, the infected or necrotic bone was removed radically. **c** Bone cement spacer. **d** External fixation. **e** Bone union was achieved at 19 months after second-stage operation. **f** Patient had removed the external fixator

The PT and IT techniques were similar in debridement. The patient remained supine on a transmissive operating table. It was ensured that the surgical incision was as consistent as possible with the previous incision, including the sinus. Hardware such as steel plates, screws, or intramedullary nails used in the previous surgery was removed. Then, the infected bone was completely removed until the so-called “paprika sign” (cortical bone bleeding area) appeared (Fig. 1a, b) [19]. It is of great importance to ensure that the cortex remaining after radical debridement is intact and uninfected.

In group A (PT), the bone defect was measured in the first stage after radical debridement and was filled with a polymethylmethacrylate (PMMA) bone cement spacer (Fig. 1c). We tended to use 4.0 g vancomycin + 4.0 g meropenem per 40 g cement. The types of antibiotics can be adjusted according to the results of bacterial culture and drug sensitivity tests before surgery. The fixation method depends on the type and location of the bone infection defect. Choices include monolateral or circular external fixations (Fig. 1d). The cement spacer was carefully removed without damaging the PMMA-induced membrane 6–8 weeks after the first operation. Next, we used an external fixation extender for comparison of the installation. Under the control of an image intensifier, two or three suitable hydroxyapatite-covered pins were used to fix the femoral or tibial shaft, which were interposed through a pre-drilled path about 2–3 cm above and below the osteotomy site. A subperiosteal transverse osteotomy was performed then. After suturing the periosteum, the incision was closed using a drainage tube. In group B (PT), after debridement and removal of the infected or necrotic bone, we chose external fixation for the tibial and femoral bone defects. The transverse osteotomy method was similar to that in group A.

In both groups, we used an open dressing change or vacuum sealing drainage (VSD) to temporarily seal large and complex soft tissue defects at the site of infection. Whether skin grafts or flaps are needed before bone defect repair depends on the condition of the soft tissue.

### Postoperative protocols

**Fig. 2:**
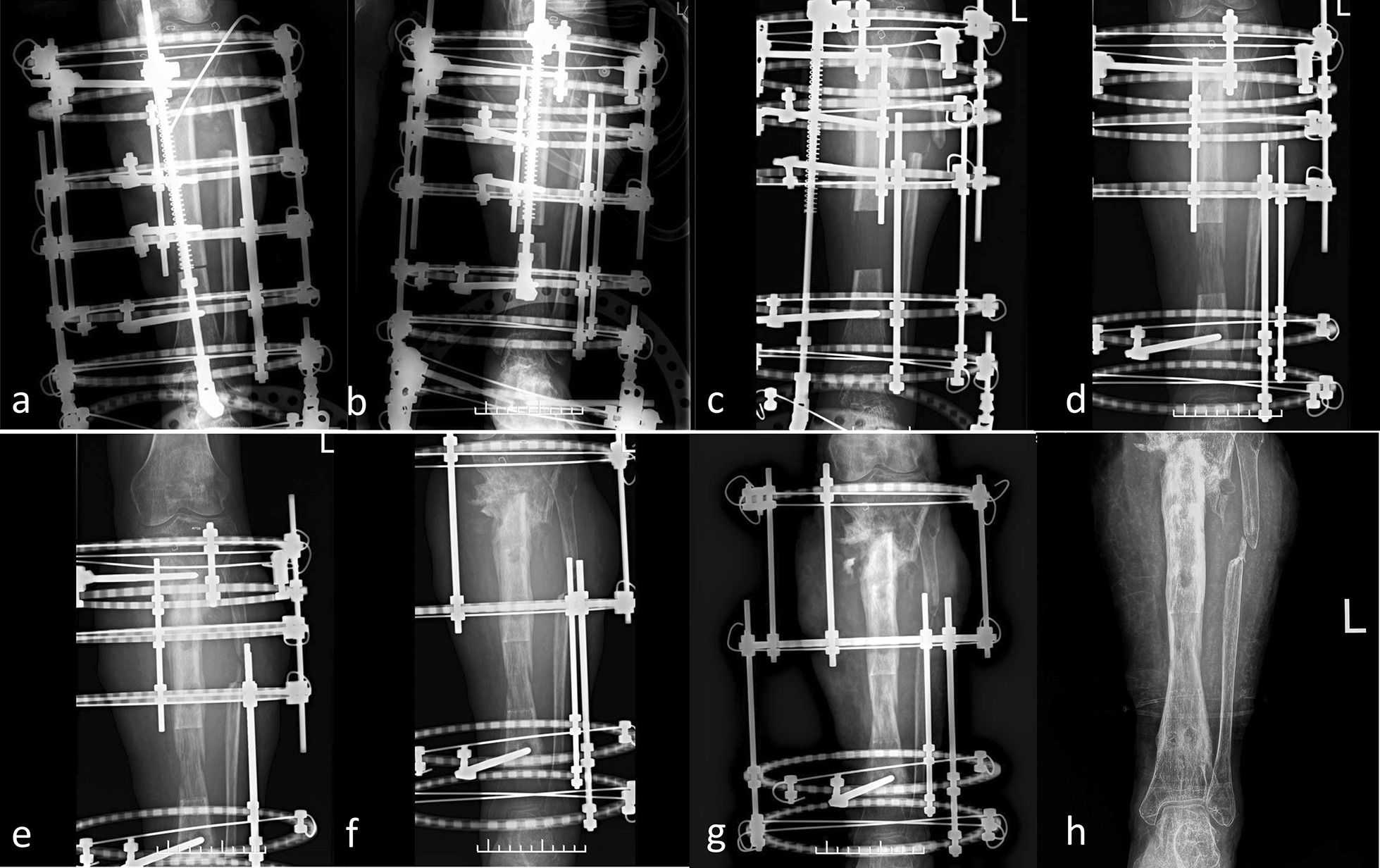
The bone transport process of a 50-year-old woman who had an infected left tibia bone defect with traditional Ilizarov technique. **a** Radiograph of a 50-year-old woman who had an infected left tibia bone defect with the start of transverse osteotomy. **b** Three weeks after operation. **c** Two months after operation. **d**–**f** Having the regenerated bone begun to be mineralized at the docking site at 7, 9 months and 1 year after operation. **g** Good consolidation and mineralization of the regenerated bone at 17 months after operation. **h** Completing bone union and removing the external fixation at 24 months after operation

Clinical follow-up was conducted every 2 weeks to check the status of the nail path, skeleton stability, range of motion and damage to adjacent joints. In the distraction phase, X-ray examination was performed every 2 weeks, followed by once a month during the consolidation period, to assess the fracture healing and the quality of consolidation (Fig. 2). Radiographic measurements were performed by two surgeons independently. Laboratory indicators, such as erythrocyte sedimentation rate (ESR), C-reactive protein (CRP), and blood cell count, were tested at appropriate time points to ensure eradication of the infection. Postoperative complications were also recorded. Dahl’s grading was used to assess pin-tract inflammation [20]. The results of bone healing and functional recovery were evaluated according to the modified Application of Methods of Illizarov (ASAMI) criteria (Table 1) [21]. The external fixation time (EFT) represents the number of whole days during which the external fixation was fixed to the bone. The external fixation index (EFI) was defined as EFT (days) divided by the total length (cm).

**Table 1:** The criteria for the assessment of bone and function 13

Bone results	Criteria	Functional results	Criteria
Excellent	Union, no infection, deformity < 7°, limb-length discrepancy (LLD) < 2.5 cm	Excellent	Active, no limp, minimum stiffness (loss of < 15° knee extension/< 15° ankle dorsiflexion), no reflex sympathetic dystrophy (RSD), insignificant pain
Good	Union plus any two of the following: absence of infection, deformity < 7°, LLD < 2.5 cm	Good	Active, with one or two of the following: limp, stiffness, RSD, significant pain
Fair	Union plus any one of the following: absence of infection, deformity < 7°, LLD < 2.5 cm	Fair	Active, with three or all of the following: limp, stiffness, RSD, significant pain
Poor	Nonunion/refracture/union plus infection plus deformity > 7° plus LLD > 2.5 cm	Poor	Inactive (unemployment or inability to return to daily activities because of injury)

### Statistical analysis

Data were analyzed using SPSS 22.0 software (SPSS Inc, Chicago, Illinois). The Kolmogorov–Smirnov test was used to check for normality. A *p* value of > 0.05 was defined as a normal distribution. The internal consistency test (kappa value) was used to assess interobserver variability in the measurement of the parameters. Descriptive statistics were used for all the variables. Continuous variables were expressed as means and standard deviations. Dichotomous variables were expressed as percentages and events. The independent samples t-test, chi-square test, or Wilcoxon rank sum test were performed to evaluate the differences between the two groups. Statistical significance was set at *p* < 0.05.

## Results

The most common cause of infection was orthopedic implant-related infection, with only a few patients suffering from open fracture and primary hematogenous infection. Both groups were most infected with Staphylococcus aureus. Detailed intraoperative and postoperative data of groups A and B are shown in Tables 2 and 3, respectively. In both groups, complete eradication of the infection and union of docking sites were accomplished. The mean bone defect length and EFT did not differ between groups A and B, while the mean external fixator index (EFI) of group A (42.32 days/cm) was significantly lower than that of group B (58.85 days/cm) (*p* < 0.001).

**Table 2:** Basic patient data of PT and IT groups

Items	PT group	IT group	*p* value
Number	12	29	–
Sex ratio (males/females)	11/1	27/2	0.657
Mean age	44.00 ± 11.73	43.17 ± 15.75	0.871
Mean times of previous operations	2.67 ± 1.15	2.52 ± 1.06	0.690
Site of injury (femur/tibia)	2/10	3/26	0.620
Right/left	4/8	17/12	0.181
Type of external fixator (circular/monolateral)	8/4	22/7	0.701

**Table 3:** Comparisons of follow-up data between PT and IT groups

Items	PT group	IT group	*p* value
Duration of cementation (days)	64	–	–
Bone defect length (cm)	9.96 ± 3.10	7.5 ± 3.04	0.024
External fixator time (days)	425.92 ± 166.35	430.90 ± 165.85	0.931
External fixator index (EFI = days/cm)	42.32 ± 8.31	58.85 ± 13.53	< 0.001
Cases of bone regraft	0	3	–
Follow-up (months)	28.50 ± 8.23	29.90 ± 8.21	0.623

According to the bone healing and limb function evaluation criteria recommended by ASAMI, the results of bone healing in group A, in which nine cases were classified as excellent and three cases as good, were similar to those in group B, which had 22 excellent cases and seven good cases (*p* = 0.558). The limb function in group A was significantly better that in group B (*p* < 0.05). Group A comprised seven excellent cases, four good cases, and one poor case. However, group B had six excellent cases, 12 good cases, 10 fair cases, and one poor case. Although there was a statistical difference between groups A and B in the functional results, there was no statistical difference in the bone results (Table 4). The relatively worse functional result was mainly due to prolonged external fixation, leading to significant pain and restricted movement of the adjacent joints.

**Table 4:** Evaluation of the bone and functional results between PT and IT groups

Outcomes	Treatment	Numbers (femur/tibia/percentage)				Total	*p* values
		Excellent	Good	Fair	Poor		
Bone results	PT group	9 (1/8)	3 (1/2)	0 (0/0)	0 (0/0)	2/10	*p* = 0.558
		75.0%	25.0%	0%	0%		
	IT group	19 (1/18)	10 (2/8)	0 (0/0)	0 (0/0)	3/26	
		65.5%	34.5%	0%	0%		
Functional results	PT group	7 (0/7)	4 (1/3)	0 (0/0)	1 (1/0)	2/10	*p* = 0.020
		58.3%	33.3%	0%	8.3%		
	IT group	6 (0/6)	12 (1/11)	10 (2/8)	1 (0/1)	3/26	
		20.7%	41.4%	34.5%	3.4%		

Pain was the most common complaint during the follow-up period. Some patients experienced severe and intolerable pain in the first few days after surgery. In both groups A and B, almost all patients had dull pain during the distraction phase, especially at night. Most patients were treated with oral analgesics to relieve the pain. In the PT group, none had delayed healing of the joint, while the IT group had three cases. They were admitted to the hospital for bone regraft and adjustment of the external fixation stent, resulting in the union of the docking sites. Grade II and III pin tract infections according to Dahl’s classification were detected in one patient in group A and six patients in group B. These patients were administered oral broad-spectrum antibiotics and local injections. Grade IV or uncontrolled infection was not observed. Although three patients in group A and eight in group B experienced transient adjacent joint stiffness when removing the fixator, all of them achieved normal range of motion in the adjacent joint after 2–3 months of rehabilitation physical training.

## Discussion

Treatment of lower limb osteomyelitis remains a serious problem in orthopedics. Some measures can be effective in preventing postoperative infection of the lower limbs, such as intramedullary antibiotic-coated nails [22]. However, infection cannot be completely avoided. Patients who decide to undergo limb salvage treatment usually require multiple surgical treatments, resulting in soft tissue and bone defects. Currently, there are many methods for the treatment of bone defects, including the Masquelet and Ilizarov techniques. In recent years, the Masquelet technique has gradually become the main method for the treatment of bone defects after lower limb infection owing to the significant advantage of the induced membrane [23, 24]. However, for large segments of bone defects, the classic Masquelet technique cannot obtain a sufficient amount of autologous bone graft and is also prone to complications at the donor site. Furthermore, it may not correct limb length discrepancy (LLD) and limb alignment. The traditional Ilizarov technique has several advantages. Patients can bear weight early after surgery. In addition, IT has the advantages of continuous adjustment of limb alignment with external fixation, and the possibility of a one-stage operation. Unfortunately, the long duration of the external fixator places restrictions on its use and can also result in many complications. There is no doubt that if the external fixation time can be effectively reduced, this technique will become very attractive. The present study investigated a novel hybrid PT combining induced membrane and bone transport techniques to effectively reduce the duration of external fixation and complications.

Our study demonstrated that the average EFI in the PT group was significantly lower than that in the IT group, and the bone healing results were not negatively affected. Traditional studies on the management of infected nonunion with Ilizarov external fixator reported an average EFI of 54.9 and 54.0 days/cm [25, 26]. The characteristics of the induced membrane can be described as a vascularized structure that resembles the periosteum, secreting vascular inducible factors (VEGF) [27, 28], and a maturity stage between the 4th and 6th weeks [29]. One of the advantages of the PT is that the blood supply of the induced membrane in the bone defect area and debridement area can promote the migration of the docking sites, thus accelerating the process of distraction osteogenesis.

Delayed union or nonunion of the docking site may occur when the contact area is insufficient or when the docking site does not coincide. Several feasible options have been proposed to prevent this common complication and shorten the duration of the fixator. Some surgeons choose to perform partial autogenous bone grafting, while some prefer the osteotome and curette to polish the bony edges at the docking site [30, 31]. Bifocal bone transport has also been reported to be effective [32]. However, our PT provides an exceptional osteogenic microenvironment compared to the IT, where the induced membrane can secrete osteoinductive factors and stimulate bone regeneration. In our study, the PT group had no case of delayed docking site union, while the IT group had three cases requiring further operations for the bone graft.

Removal of the entire bone segment and continuous antibiotic release with bone cement can effectively remove the dead bone and biofilm, thus offering a better chance of eliminating infection compared to the Ilizarov technique. Although no infection recurrence was noted in either group, Roukoz et al. [33] and Shah et al. [34] have reported that antibiotic-loaded bone cement can achieve a better anti-infection effect. A narrative review [35] showed that the rate of infection recurrences in patients with infected or non-infected critical-sized tibial bone defects treated using the Ilizarov method was 4.58%. Although there was no significant difference in the operation times between the two groups, a logistic regression analysis [36] revealed that repeated operations, post-traumatic osteomyelitis, and internal fixation in the first stage were risk factors for recurrence of infection in patients treated with the induced membrane technique. Based on these concerns, we recommend that repeat operations and the surgical design of internal fixation should be carefully considered to allow patients to receive more benefits from PT.

Although the PT requires a two-stage operation, its advantages outweigh the disadvantages. Owing to the potential benefits of the induced membrane, rich blood supply, and satisfactory union of the docking site, the PT can gather remarkably reduced EFI, satisfactory bone healing results, and better functional results. In addition, early removal of the external fixator could significantly reduce the risk of complications. In the PT group, the percentage and severity of pain, joint stiffness and pin tract infection were low, and no complications occurred that required further surgical treatment.

## Conclusions

The limitations of our study are the relatively small sample size and short follow-up period. Nevertheless, the PT provides an early opportunity to remove the fixator, allowing for earlier rehabilitation exercises and eliminating patient discomfort. We believe that this new combined technique provides a feasible and improved method for the treatment of patients with bone defects after lower extremity infection.

## Supplementary Information

**Additional file 1**. The datasets of patients.

## Abbreviations

PT: Piston technique
IT: Ilizarov technique
ASAMI: Association for the Study and Application of the Methods of Illizarov
EFI: External fixator index
EFT: External fixator time
PMMA: Polymethylmethacrylate
VSD: Vacuum sealing drainage
ESR: Erythrocyte sedimentation rate
CRP: C-reactive protein

## References

[1] Kanakaris NK, Tosounidis TH, Giannoudis PV (2015). Surgical management of infected non-unions: an update. Injury.

[2] Yang KH, Won Y, Kim SB, Oh BH, Park YC, Jeong SJ (2016). Plate augmentation and autologous bone grafting after intramedullary nailing for challenging femoral bone defects: a technical note. Arch Orthop Trauma Surg.

[3] Aronson J, Johnson E, Harp JH (1989). Local bone transportation for treatment of intercalary defects by the Ilizarov technique. Biomechanical and clinical considerations. Clin Orthop Relat Res.

[4] Demiralp B, Ege T, Kose O, Yurttas Y, Basbozkurt M (2014). Reconstruction of intercalary bone defects following bone tumor resection with segmental bone transport using an Ilizarov circular external fixator. J Orthop Sci.

[5] Madhusudhan TR, Ramesh B, Manjunath K, Shah HM, Sundaresh DC, Krishnappa N (2008). Outcomes of Ilizarov ring fixation in recalcitrant infected tibial non-unions—a prospective study. J Trauma Manag Outcomes.

[6] Dendrinos GK, Kontos S, Lyritsis E (1995). Use of the Ilizarov technique for treatment of non-union of the tibia associated with infection. J Bone Joint Surg Am.

[7] Selhi HS, Mahindra P, Yamin M, Jain D, De Long WG, Singh J (2012). Outcome in patients with an infected nonunion of the long bones treated with a reinforced antibiotic bone cement rod. J Orthop Trauma.

[8] Keating JF, Simpson AH, Robinson CM (2005). The management of fractures with bone loss. J Bone Joint Surg Br.

[9] Taylor GI, Miller GD, Ham FJ (1975). The free vascularized bone graft. A clinical extension of microvascular techniques. Plast Reconstr Surg.

[10] Urist MR, Sato K, Brownell AG, Malinin TI, Lietze A, Huo YK (1983). Human bone morphogenetic protein (hBMP). Proc Soc Exp Biol Med.

[11] Lin CC, Chen CM, Chiu FY, Su YP, Liu CL, Chen TH (2012). Staged protocol for the treatment of chronic tibial shaft osteomyelitis with Ilizarov's technique followed by the application of intramedullary locked nail. Orthopedics.

[12] Khan MS, Rashid H, Umer M, Qadir I, Hafeez K, Iqbal A (2015). Salvage of infected non-union of the tibia with an Ilizarov ring fixator. J Orthop Surg (Hong Kong).

[13] Masquelet AC, Fitoussi F, Begue T, Muller GP (2000). Reconstruction of the long bones by the induced membrane and spongy autograft. Ann Chir Plast Esthet.

[14] Careri S, Vitiello R, Oliva MS, Ziranu A, Maccauro G, Perisano C (2019). Masquelet technique and osteomyelitis: innovations and literature review. Eur Rev Med Pharmacol Sci.

[15] Lalidou F, Kolios G, Drosos GI (2014). Bone infections and bone graft substitutes for local antibiotic therapy. Surg Technol Int.

[16] van Vugt TAG, Arts JJ, Geurts JAP (2019). Antibiotic-loaded polymethylmethacrylate beads and spacers in treatment of orthopedic infections and the role of biofilm formation. Front Microbiol.

[17] Rajendran M, Iraivan G, Ghayathri B, Mohan P, Chandran KR, Nagaiah H (2020). Antibiotic loaded nano rod bone cement for the treatment of osteomyelitis. Recent Pat Nanotechnol.

[18] Tong K, Zhong Z, Peng Y, Lin C, Cao S, Yang Y (2017). Masquelet technique versus Ilizarov bone transport for reconstruction of lower extremity bone defects following posttraumatic osteomyelitis. Injury.

[19] Eralp L, Kocaoglu M, Rashid H (2007). Reconstruction of segmental bone defects due to chronic osteomyelitis with use of an external fixator and an intramedullary nail. Surgical technique. J Bone Joint Surg Am.

[20] Dahl MT, Gulli B, Berg T (1994). Complications of limb lengthening. A learning curve. Clin Orthop Relat Res.

[21] Paley D, Catagni MA, Argnani F, Villa A, Benedetti GB, Cattaneo R (1989). Ilizarov treatment of tibial nonunions with bone loss. Clin Orthop Relat Res.

[22] Greco T, Vitiello R, Cazzato G, Cianni L, Malerba G, Maccauro G (2020). Intramedullary antibiotic coated nail in tibial fracture: a systematic review. J Biol Regul Homeost Agents.

[23] Zhang Q, Zhang W, Zhang Z, Zhang L, Chen H, Hao M (2017). Femoral nonunion with segmental bone defect treated by distraction osteogenesis with monolateral external fixation. J Orthop Surg Res.

[24] van Niekerk AH, Birkholtz FF, de Lange P, Tetsworth K, Hohmann E (2017). Circular external fixation and cemented PMMA spacers for the treatment of complex tibial fractures and infected nonunions with segmental bone loss. J Orthop Surg (Hong Kong).

[25] Kocaoglu M, Eralp L, Rashid HU, Sen C, Bilsel K (2006). Reconstruction of segmental bone defects due to chronic osteomyelitis with use of an external fixator and an intramedullary nail. J Bone Joint Surg Am.

[26] Sigmund IK, Ferguson J, Govaert GAM, Stubbs D, McNally MA (2020). Comparison of Ilizarov bifocal, acute shortening and relengthening with bone transport in the treatment of infected, segmental defects of the tibia. J Clin Med.

[27] Pelissier P, Masquelet AC, Bareille R, Pelissier SM, Amedee J (2004). Induced membranes secrete growth factors including vascular and osteoinductive factors and could stimulate bone regeneration. J Orthop Res.

[28] Cuthbert RJ, Churchman SM, Tan HB, McGonagle D, Jones E, Giannoudis PV (2013). Induced periosteum a complex cellular scaffold for the treatment of large bone defects. Bone.

[29] Wang X, Wei F, Luo F, Huang K, Xie Z (2015). Induction of granulation tissue for the secretion of growth factors and the promotion of bone defect repair. J Orthop Surg Res.

[30] Emara KM, Ghafar KA, Al Kersh MA (2011). Methods to shorten the duration of an external fixator in the management of tibial infections. World J Orthop.

[31] Sala F, Thabet AM, Castelli F, Miller AN, Capitani D, Lovisetti G (2011). Bone transport for postinfectious segmental tibial bone defects with a combined Ilizarov/Taylor spatial frame technique. J Orthop Trauma.

[32] Griffith MH, Gardner MJ, Blyakher A, Widmann RF (2007). Traumatic segmental bone loss in a pediatric patient treated with bifocal bone transport. J Orthop Trauma.

[33] Roukoz S, El Khoury G, Saghbini E, Saliba I, Khazzaka A, Rizkallah M (2020). Does the induced membrane have antibacterial properties? An experimental rat model of a chronic infected nonunion. Int Orthop.

[34] Shah SR, Smith BT, Tatara AM, Molina ER, Lee EJ, Piepergerdes TC (2017). Effects of local antibiotic delivery from porous space maintainers on infection clearance and induction of an osteogenic membrane in an infected bone defect. Tissue Eng Part A.

[35] Aktuglu K, Erol K, Vahabi A (2019). Ilizarov bone transport and treatment of critical-sized tibial bone defects: a narrative review. J Orthop Traumatol.

[36] Wang X, Wang S, Fu J, Sun D, Shen J, Xie Z (2020). Risk factors associated with recurrence of extremity osteomyelitis treated with the induced membrane technique. Injury.

